# Prevalence and Molecular Analysis of Encephalomyocarditis Virus-2 in the Hazel Dormouse

**DOI:** 10.1007/s10393-024-01680-z

**Published:** 2024-04-23

**Authors:** Louise Gibson, Tammy Shadbolt, Pranab Paul, Georgina Gerard, Ethan Wrigglesworth, Anthony W. Sainsbury, Helen Donald, Jenny E. Jaffe, Inez Januszczak, Liam D. Fitzpatrick, Caela Burrell, Hannah Davies, Akbar Dastjerdi, Simon Spiro

**Affiliations:** 1 Institute of Zoology, Zoological Society of London, London, NW1 4RY UK; 2 Royal Veterinary College, London, UK; 3 Chattogram Veterinary and Animal Sciences University, Chittagong, Bangladesh; 4 Natural England, London, UK; 5 Tai Chimpanzee Project, Abidjan, Côte d’Ivoire; 6 Natural History Museum, London, UK; 7 UK Health Security Agency, London, UK; 8 Animal and Plant Health Agency-Weybridge, Surrey, UK

**Keywords:** Encephalomyocarditis virus, EMCV-2, *Cardiovirus*, Hazel dormouse, *Muscardinus avellanarius*

## Abstract

The hazel dormouse (*Muscardinus avellanarius*) population in the UK continues to decline due to habitat loss, despite reintroductions of captive-bred individuals being conducted nationally for over 30 years. Disease surveillance of captive-bred and wild dormice is performed to identify novel and existing disease threats which could impact populations. In this study, we firstly investigated cause of death in seven hazel dormice found dead in England, through next-generation sequencing identifying a virus closely related to a wood mouse encephalomyocarditis virus-2 (EMCV-2). Subsequently, lung tissue samples from 35 out of 44 hazel dormice tested positive for EMCV-2 RNA using a reverse transcriptase quantitative polymerase chain reaction (RT-qPCR) and Sanger sequencing methods developed in this study. Formalin-fixed tissues available for nine hazel dormice which tested positive for EMCV-2 RNA were examined microscopically. Three cases showed moderate interstitial pneumonia with minimal to mild lymphoplasmacytic myocarditis, but no evidence of encephalitis. However, the presence of possible alternative causes of death in these cases means that the lesions cannot be definitively attributed to EMCV-2. Here, we report the first detection of EMCV-2 in hazel dormice and conclude that EMCV-2 is likely to be endemic in the hazel dormouse population in England and may be associated with clinical disease.

**Supplementary Information:**

The online version contains supplementary material available at 10.1007/s10393-024-01680-z.

## Introduction

The hazel dormouse (*Muscardinus avellanarius*) in the UK is a protected species as they are vulnerable to extinction due to a small population size. It is a priority species in the UK Biodiversity Action Plan and protected under the Wildlife and Countryside Act 1981. The population in the UK has declined by 72% since 1993 which is believed to be due to loss of suitable woodland habitat (Goodwin et al. 2017). A collaborative initiative between Natural England (NE), Common Dormouse Captive Breeders Group (CDCBG), Zoological Society of London (ZSL) and the People’s Trust for Endangered Species (PTES) has facilitated the release of 1029 captive-bred hazel dormice into managed English woodlands in 13 counties since 1993 with the aim of supplementing and restoring their population (People’s Trust for Endangered Species 2024). Each year juvenile captive-bred dormice are transferred to bio-secure units for a 10-week quarantine period and health screened by a wildlife veterinarian. Individuals free from clinical signs of disease and testing negative for suspected alien faecal parasites are considered fit for release. Pre-release health screening thus mitigates against the probability of inadvertently introducing novel infectious agents into the free-living population. Continuous health surveillance of the species is critical for identifying unknown disease threats which could impact populations as well as informing pre-release screening. The Disease Risk Analysis and Health Surveillance (DRAHS) project at ZSL conducts post-mortem examinations of hazel dormice found dead in their natural habitat or submitted from conservation centres involved in breeding for the reintroduction programme. Common causes of mortality we identify at post-mortem examination include traumatic injuries due to predation, systemic bacterial infections or failure to thrive in newborn young.

Encephalomyocarditis virus (EMCV) is a small, non-enveloped, positive-sense single-stranded RNA in the *Cardiovirus* genus of the *Picornaviridae* family. EMCV-1 was first identified in 1945 from a captive gibbon in Florida that died suddenly with pulmonary oedema and myocarditis (Helwig and Schmidt 1945). EMCV-1 has since been detected in at least 30 host species worldwide, including domestic dogs, cats, horses, cows, pigs, free-living rodents, non-human primates, elephants, raccoons, mongoose and pheasants (Billinis 2009; Carocci and Bakkali-Kassimi 2012; Philipps et al. 2012; Qin et al. 2018). Rodents are considered the natural, primary reservoir hosts and vectors of the virus (Seaman et al. 1986), and faecal–oral transmission is considered to occur through the ingestion of contaminated food or water (Zimmerman et al. 2019). EMCV-1 infection may be asymptomatic in rodent species; however, it has also been associated with disease (Cerutis et al. 1989). EMCV-1-induced myocarditis with progression to heart failure has been documented in laboratory mice (Higuchi et al. 2008). In addition, EMCV-1 has been isolated from two clinically ill free-living edible dormice (*Myoxus glis*) in different regions of Italy (Amaddeo et al. 1995). EMCV-2 was first isolated from a captive wood mouse (*Apodemus sylvaticus*) in Germany and described in 2012 (Philipps et al. 2012). Four member viruses of the *Cardiovirus A* species have since been proposed: EMCV-1-4; however, the host range and pathogenicity of recently isolated EMCV-2-4 remain undetermined (Vyshemirskii et al. 2018).

Post-mortem examination findings obtained through the DRAHS project are regularly reviewed in order to identify case commonalities which may indicate a novel disease threat. Additionally, non-targeted diagnostics such as bacterial culture, faecal parasitology and next-generation sequencing (NGS) may be performed in order to understand which pathogen or potential pathogens may be present in the populations. However, such techniques alone cannot inform whether these agents are causing disease.

In this study, we report the first detection of EMCV-2 in hazel dormice by NGS in a pooled sample of lung tissue from seven dormouse carcases submitted to the DRAHS project for post-release health surveillance. We further report the first preliminary evidence of virus presence in the wider population of dormice by screening the carcases of all suitably well-preserved hazel dormice submitted for post-mortem examination between 2019 and 2022 (44 animals) using a reverse transcriptase quantitative polymerase chain reaction (RT-qPCR) developed in this study. We also report gross and histopathological findings from nine positive animals and suggest that EMCV-2 infection in hazel dormice is unlikely to be associated with lesions.

## Materials and Methods

### Sample Collection

Carcases of dormice found dead in England opportunistically by members of the public or wildlife officers had been submitted to the DRAHS project for post-mortem examination as part of ongoing disease surveillance for the species. Carcases were either examined immediately or frozen at − 20 °C and examined at a later date. During the examination, adequately preserved tissues were sampled into 10% neutral buffered formalin and lung tissue preserved frozen at − 80 °C.

### Next-Generation Sequencing

Lung tissue samples from seven dormice collected during post-mortem examinations between 2016 and 2017 and preserved frozen at − 80 °C were homogenised in 300 μl PBS using the gentleMACS Dissociator (Miltenyi Biotec) and centrifuged at 5000 g for 3 min, and nucleic acid (RNA and DNA) was extracted from 140 μl of supernatant using QIAamp Viral RNA mini kit (Qiagen) according to the manufacturer’s instructions, but excluding the carrier RNA. The extracted nucleic acids, without random amplification, were pooled and subjected to complementary DNA (cDNA) synthesis using SuperScript™ IV First-Strand Synthesis System (Thermo Fisher Scientific) and NEBNext^®^ Ultra II Non-Directional RNA Second Strand Synthesis Module (New England Biolabs). DNA concentration was then measured using a fluorescent DNA-binding dye before proceeding to library preparation. The library preparation was done using the small whole-genome—Nextera XT kit (Illumina)—following the manufacturer’s instructions. Double-stranded DNA was randomly broken into small fragments (typically less than 500 bp) as part of the library preparation, and the libraries were assessed again for concentration and combined in an equimolar pool. NGS was carried out on an Illumina NextSeq instrument.

Raw NGS data were initially screened against viral reference sequences (RefSeq) from GenBank using SeqMan NGen17.5 (DNASTAR) metagenomics pipeline and reference-guided option; assembly was set at minimum match of 50%. The Raw NGS data subsequently mapped to hazel dormouse genome GenBank accession number GCA_004027005 as above and unassembled sequence reads were subjected to de novo assembly using de novo option of the metagenomic pipeline. Finally, wood mouse EMCV-2 sequence, GenBank accession number JX257003, was used to assemble the dormouse EMCV-2 genome sequence.

### Nucleic Acid Extraction, Real-Time RT-qPCR Screening and RT-PCR

Lung tissue samples from the additional 44 hazel dormice collected during post-mortem examinations between 2019 and 2022 and preserved frozen at − 80 °C were homogenised in lysis buffer using a micro-pestle. Viral RNA was extracted using QIAamp Viral RNA mini kit (Qiagen) according to the manufacturer’s instructions to produce a 50 µl RNA elution. The RNA elution was DNase treated using TURBO DNA-free^™^ Kit (Invitrogen) according to manufacturer’s instructions.

EMCV-2 primers and probe for RT-qPCR were designed using Primer3 version 4.1.0 (Rozen and Skaletsky 2000) (Table 1). Real-time RT-qPCR was performed using the QuantiFast Pathogen RT-PCR + IC Kit (Qiagen). Each 25 µl RT-qPCR contained 10.75 µl RNase-Free water, 5 µl of 5 × QuantiFast Pathogen Master Mix, 0.25 µl QuantiFast Pathogen RT Mix, 0.5 µl of 50 × High-ROX Dye Solution, 2.5 µl Internal Control Assay, 2.5 µl Internal Control RNA, 0.5 µl of 10 µM EMCV-2Q F, 0.5 µl of 10 µM EMCV-2Q R, 0.5 µl of 5 µM EMCV-2Q P and 2 µl of extracted RNA template. Thermocycling conditions were carried out at 50 °C for 20 min, 95 °C for 5 min followed by 45 cycles of 95 °C for 15 s and 60 °C for 45 s before being held at 4 °C. Negative extraction and no template controls were included in each RT-qPCR run to ensure lack of cross-contamination during the extraction and RT-qPCR steps, respectively. An internal RT-qPCR control provided in the QuantiFast Pathogen RT-PCR + IC Kit was also added to each sample to check for RT-qPCR efficiency and inhibitors. A serial dilution of synthetic EMCV-2 DNA was also included in each RT-qPCR run as a positive RT-qPCR control and to generate a standard curve.

**Table 1:** Forward and Reverse Primers Used for RT-qPCR and Conventional RT-PCR Detection of Dormice EMCV-2.

Primer	Primer sequence (5′-3′)	Amplicon size
EMCV-2Q F	TCTAGCAAAGACAGGGTAC	131 bp
EMCV-2Q R	TGACAGGACGATACAAG	
EMCV-2Q P	FAM-TCCTCTTGAGTCTACTTTGGCAGA-BHQ1	
EMCV-2A F	GTGCTGCCACTTCAATGTT	
EMCV-2A R	AGCCGATCATATTCCTCCTT	379 bp

Suffix F; forward primer, P; probe, R; reverse primer.

EMCV-2 primers for conventional RT-PCR and sequencing were designed using Primer 3 version 4.1.0 (Rozen and Skaletsky 2000) (Table 1). EMCV-2 RT-qPCR-positive samples were amplified for DNA sequencing using SuperScript™ III One-Step RT-PCR System with Platinum™ Taq DNA Polymerase kit (Invitrogen). Each 25 µl RT-PCR contained 6 µl RNase-free water, 12.5 µl of 2 × Reaction Mix, 1.5 µl of 10 µM EMCV-2A F primer, 1.5 µl of 10 µM EMCV-2A R primer, 0.5 µl of 10 µM dNTPs, 1 µl of SuperScript III RT/Platinum Taq Mix and 2 µl of extracted RNA template. Thermocycling conditions were carried out at 50 °C for 30 min, 95 °C for 15 min followed by 40 cycles of 94 °C for 30 s, 60 °C for 30 s, 72 °C for 1 min and a final extension of 72 °C for 10 min before being held at 4 °C.

**Figure 1:**
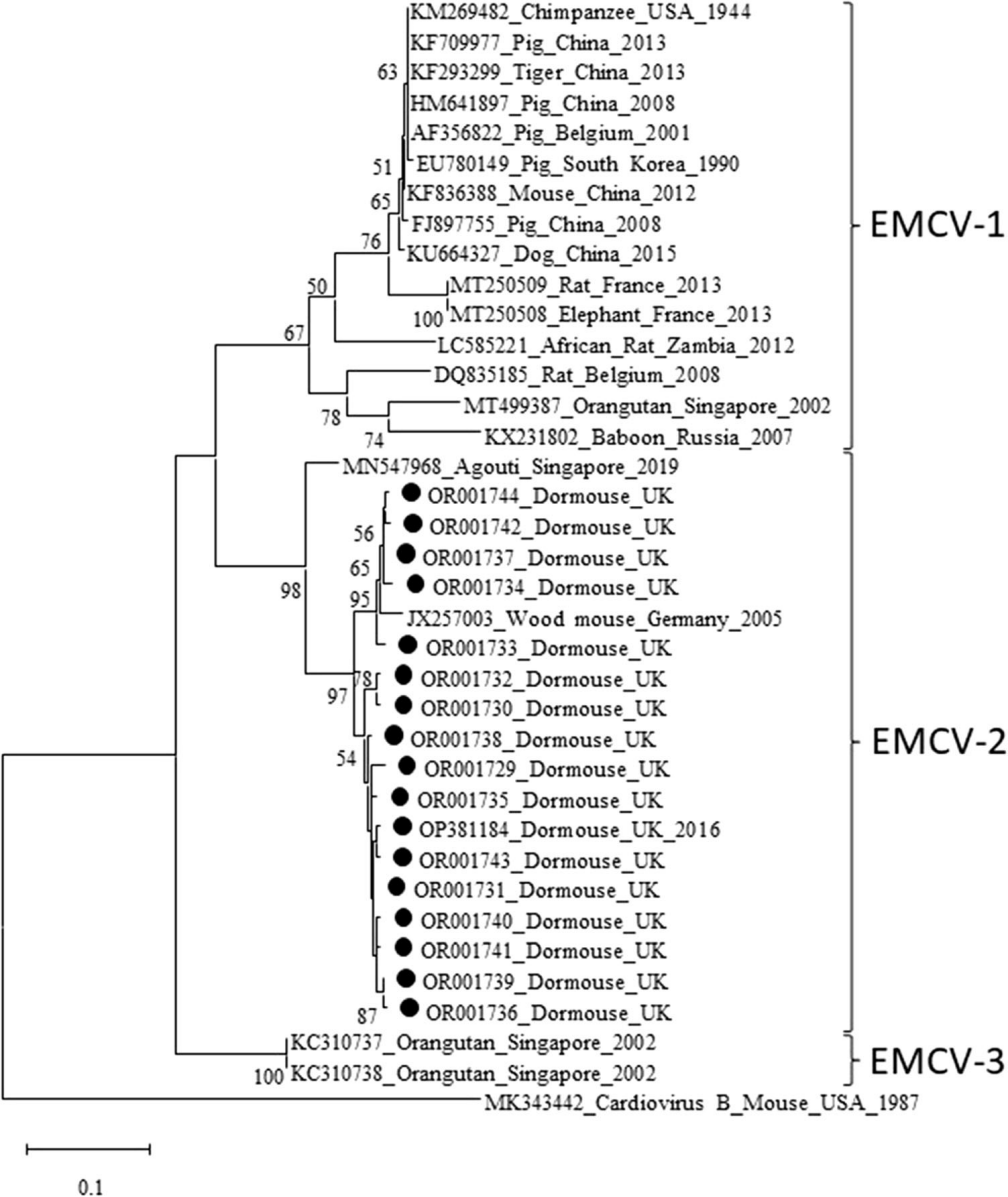
Phylogenetic analysis of a 379-bp DNA fragment of the encephalomyocarditis virus 3D polymerase gene. The DNA sequences were aligned with MUSCLE and the alignment was subjected to phylogenetic analysis using MEGA X software. The evolutionary history was inferred by using the maximum likelihood method, 500 bootstrap replicates and Tamura–Nei model (Tamura et al., 2021). The tree with the highest log likelihood (-2519.94) is shown. The percentage of trees in which the associated taxa clustered together is shown next to the branches. Initial tree(s) for the heuristic search were obtained automatically by applying Neighbor-Join and BioNJ algorithms to a matrix of pairwise distances estimated using the maximum composite likelihood (MCL) approach and then selecting the topology with superior log likelihood value. The tree is drawn to scale, with branch lengths measured in the number of substitutions per site. *Cardiovirus B,* GenBank accession number MK343442 was used as an outgroup. Bootstrap values above 50% are only shown in the tree.

PCR products were resolved by 2% (w/v) agarose gel electrophoresis followed by visualisation using GelGreen (Biotium) nucleic acid stain and blue light. Amplicons of the anticipated size (Table 1) were purified using a QIAquick Gel Purification Kit (Qiagen) following manufacturer’s instructions and were Sanger sequenced by Eurofins Genomics. Sequences were analysed using Geneious 7.1.9 (Kearse et al. 2012), primers trimmed and aligned with those of GenBank published EMCV-1, EMCV-2 and EMCV-3 sequences using MUSCLE (Edgar 2004). A maximum likelihood phylogenetic tree was constructed in MEGA X using 500 bootstraps/replicates, rooting with *Cardiovirus B*, GenBank accession number MK343442 (Fig. 1).

### Histopathology

Samples of lung, heart, liver, kidney and brain, fixed in 10% neutral buffered formalin, were available for nine of the hazel dormice in which EMCV-2 RNA was detected by RT-qPCR. These tissues were embedded in paraffin, sectioned at 4 µm thickness, stained with haematoxylin and eosin (HE) stain using standard histological techniques (Suvarna et al. 2019) and examined by a board-certified pathologist.

## Results

### Initial Detection

NGS was performed on a pooled sample of frozen lung tissue from the post-mortem examinations of seven dormice submitted to the DRAHS project between 2016 and 17. Using GenBank viral reference sequences as a template, an EMCV was identified from the NGS data with an initial template coverage of 24.7% of the human EMCV Ruckert strain genome, RefSeq NC_001479. The de novo assembly of dormouse genome-depleted NGS reads produced 397 contigs from which three contigs, with sizes of 843, 1040 and 2817 nucleotides, showed 92.9% to 96.2% nucleotide identity to those of a wood mouse (*Apodemus sylvaticus*) EMCV-2 (GenBank accession number JX257003.1) through BLAST search (https://blast.ncbi.nlm.nih.gov/Blast.cgi) analysis. Finally, using this wood mouse EMCV-2 sequence as template, a total of 5,105 of 8,387,184 sequence reads could be assembled to create the near complete dormouse EMCV-2 genome sequence (7608 nucleotides) with a median coverage of 125.06. This sequence was deposited in GenBank under accession number OP381184. Nucleotide sequence of the 3D polymerase gene of the identified virus revealed 94% identity with that of the wood mouse EMCV-2. The original seven animals had only non-specific pulmonary lesions, such as red discolouration and/or sinking in water, and only one animal had had histopathology, which only showed agonal changes. As a result, the significance of the EMCV-2 detection in at least one of seven dormice was not clear.

### Real-Time RT-qPCR AND Phylogenetic Analysis of Conventional RT-PCR Amplicons

To investigate the commonness of EMCV-2 in the UK dormouse population, the genome sequence obtained above was used to design a real-time RT-qPCR for screening and a conventional RT-PCR for sequencing purposes. Real-time RT-qPCR detected EMCV-2 RNA in the lungs of 35 out of 44 hazel dormice. Conventional RT-PCR confirmed the presence of EMCV-2 RNA using Sanger sequencing of 379-bp amplicons of the 3D polymerase gene region. cDNA sequences were obtained from 31 RT-qPCR EMCV-2-positive samples with 16 being unique sequences (GenBank accession number OR001729- OR001744). These unique sequences (379 bp) shared high sequence identities (94.5–97.9%) with the German wood mouse EMCV-2 sequence (GenBank accession number JX257003) and 95.8–99.7% with the UK dormouse sequence (GenBank accession number OP381184). Accordingly, these EMCV-2 clustered closely in the phylogenetic tree with the wood mouse isolate, but was distinct from the previously detected EMCV-2 isolate in an agouti (GenBank accession number MN547968) and clades EMCV-1 and EMCV-3 in other animal species (Fig. 1). It was not possible to obtain sequences for four RT-qPCR-positive samples due to low viral RNA quantities, observed by high Ct values (36.3–37.6 Ct) [Supplementary table S1].

### Pathological Findings

To investigate the significance of EMCV-2 infection in dormice, histopathological examination was performed of sections of lung, heart, liver, kidney and brain from nine hazel dormice which had tested positive for EMCV-2 infection on RT-qPCR of lung tissue. In almost all cases, lungs were diffusely dark red/black and/or sank in formalin; this was consistent with autolysis and freeze/thaw artefact and, together with the small size of the tissues, prevented reliable detection and interpretation of gross lesions. Autolysis, varying from mild to marked, was present in all cases and prevented examination of certain tissues such as the gastrointestinal tract and pancreas. Other tissues such as the spleen, lymph nodes and thymus were not examined as their small size made them difficult to reliably dissect from the autolysed carcases. Of the nine cases examined, three (dormice 3, 33 and 34) had moderate, diffuse lymphoplasmacytic and neutrophilic interstitial pneumonia with concurrent minimal to mild, multifocal lymphoplasmacytic and neutrophilic myocarditis. Pure cultures of *Staphylococcus aureus* were grown from swabs of liver, lung and heart of dormouse 3, which also had moderate, multifocal lymphoplasmacytic and neutrophilic tubulointerstitial nephritis and mild, diffuse neutrophilic portal hepatitis. The other two animals (33 and 34) were orphaned siblings that were being hand-reared after the dam had died of endometritis. Cultures of peritoneal swabs from dormice 33 and 34 were negative; an abscess in the lung of dormouse 34 was presumed to be bacterial, but was not swabbed as it was not discovered until histopathology was performed. Four animals (16, 22, 33 and 34) had moderate, diffuse hepatic lipidosis, correlated either with age (neonatal juveniles) and/or with concurrent disease (negative energy balance). All other tissues, including the brains, were unremarkable.

### Demographic Data

**Figure 2:**
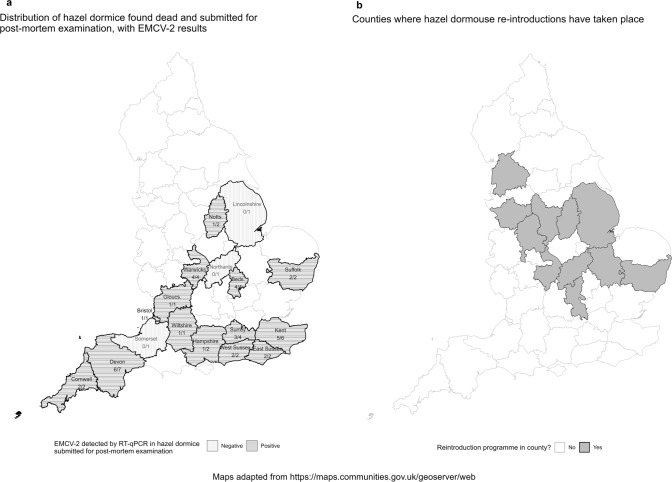
**a** Distribution of 43 hazel dormice found dead in English counties and submitted for post-mortem examination. One hazel dormouse was submitted without location data. Counties with positive and negative EMCV-2 cases, detected by RT-qPCR on lung samples, are in blue and yellow, respectively. The number of positive cases and total number of submissions from the county are also reported in the map. **b** Map of English counties with and without reintroduction in the county.

Forty-three out of the total 44 hazel dormice (Table 2) examined in this study had been submitted from 17 counties in England: Bedfordshire, Bristol, Cornwall, Devon, East Sussex, Gloucestershire, Hampshire, Kent, Lincolnshire, Northamptonshire, Nottinghamshire, Warwickshire, West Sussex, Wiltshire, Somerset, Suffolk and Surrey (Fig. 2a). One animal was submitted without location data. Twenty-nine animals were juveniles (< 6 months of age), and 15 were adults (> 6 months of age). The cohort consisted of 12 males, 12 females and 20 animals of unknown sex.

**Table 2:** Demographic data pertaining to 44 hazel dormice testing positive (POS) or negative (NEG) for EMCV-2 infection using RT-qPCR and indicating whether histopathology was undertaken.

Dormouse	Sex	Age	County	EMCV-2 RT-qPCR	Histopathology
1	Male	Juvenile	Lincolnshire	NEG	
2	Unknown	Juvenile	Hampshire	POS	
3	Male	Juvenile	Cornwall	POS	Yes
4	Male	Juvenile	Devon	POS	Yes
5	Unknown	Juvenile	Bedfordshire	POS	
6	Unknown	Juvenile	Bedfordshire	POS	
7	Unknown	juvenile	Bedfordshire	POS	
8	Unknown	juvenile	Bedfordshire	POS	
9	Male	Juvenile	Nottinghamshire	NEG	
10	Unknown	Juvenile	Northamptonshire	NEG	
11	Female	Adult	Devon	POS	
12	Male	Juvenile	Nottinghamshire	POS	
13	Male	Adult	Warwickshire	POS	
14	Female	Adult	Warwickshire	POS	
15	Male	Adult	Wiltshire	POS	
16	Female	Adult	Devon	POS	Yes
17	Female	Adult	Devon	NEG	
18	Male	Adult	Unknown	NEG	
19	Unknown	Adult	Surrey	NEG	
20	Male	Adult	Somerset	NEG	
21	Female	Adult	Suffolk	POS	
22	Female	Adult	Cornwall	POS	Yes
23	Male	Adult	Gloucestershire	POS	
24	Male	Juvenile	Bristol	POS	Yes
25	Female	Juvenile	Suffolk	POS	
26	Female	Juvenile	Devon	POS	Yes
27	Female	Adult	Devon	POS	Yes
28	Female	Juvenile	Devon	POS	
29	Unknown	Juvenile	Kent	POS	
30	Unknown	Juvenile	Kent	POS	
31	Unknown	Juvenile	West Sussex	POS	
32	Unknown	Juvenile	West Sussex	POS	
33	Unknown	Juvenile	Surrey	POS	Yes
34	Unknown	Juvenile	Surrey	POS	Yes
35	Unknown	Juvenile	Surrey	POS	
36	Unknown	Juvenile	East Sussex	POS	
37	Unknown	Juvenile	East Sussex	POS	
38	Female	Adult	Hampshire	NEG	
39	Female	Adult	Kent	POS	
40	Male	Juvenile	Kent	POS	
41	Unknown	Juvenile	Kent	NEG	
42	Unknown	Juvenile	Kent	POS	
43	Unknown	Juvenile	Warwickshire	POS	
44	Unknown	Juvenile	Warwickshire	POS

The 35 hazel dormice positive for EMCV-2 RNA by RT-qPCR were submitted from 14 counties in England: Bedfordshire, Bristol, Cornwall, Devon, East Sussex, Gloucestershire, Hampshire, Kent, Nottinghamshire, Suffolk, Surrey, Warwickshire, West Sussex and Wiltshire (Fig. 2a). EMCV-2 was detected in counties both with (Bedfordshire, Nottinghamshire, Suffolk and Warwickshire) and without (Bristol, Cornwall, Devon, East Sussex, Gloucestershire, Hampshire, Kent, Surrey and West Sussex) hazel dormouse reintroductions (Fig. 2b). Of the 35 animals, 24 were juveniles and 11 were adults; and nine were male, 10 were female, and 16 animals were of unknown sex.

The nine hazel dormice in which EMCV-2 RNA was not detected by RT-qPCR were submitted from eight counties (and one unknown location) in England: Devon, Hampshire, Kent, Lincolnshire, Northamptonshire, Nottinghamshire, Somerset and Surrey. Of these, four animals were juveniles and five were adults with a mix of four males, two females and three animals of unknown sex.

## Discussion

Here we report, to the best of our knowledge, the first evidence of EMCV-2 infection in hazel dormice, suggesting a newly identified host species for the virus. We have managed to obtain a near complete genome sequence and additional 16 distinct partial 3D polymerase gene sequences expanding the number of EMCV-2 sequences available in the GenBank database from 1 partial and 2 complete genomes (GenBank accession numbers MN547968) isolated from a greater bandicoot rat GenBank accession number MT085334 (Wu et al. 2021), a German wood mouse GenBank accession number JX257003 (Philipps et al. 2012) and an agouti GenBank accession number JX257003 (unpublished, Wang et al. 2019), respectively. Although a relatively conserved fragment of the virus genome was used for genetic analysis, the viruses show considerable genetic variations, and this warrants further research to explore the extent of their diversity and potential recombination.

EMCV-2 appears to be highly prevalent in the hazel dormouse population, with 35 out of 44 animals testing positive for the virus RNA in the lungs. We examined frozen lungs as the original detection had been made from this tissue, but organs such as brain and heart, which are the primary targets of EMCV-1, may contain higher viral loads, especially in cases with lesions and should be considered in future studies. EMCV-2, therefore, may have been present in other organs of the hazel dormice whose lung tissues tested negative for EMCV-2 RNA.

Histopathological investigations showed no evidence of pathology attributable to viral infection in six out of nine (66%) of the animals examined, positive for EMCV-2 RNA. The remaining three animals all had moderate interstitial pneumonia and minimal to mild myocarditis, both of which are lesions previously described in laboratory mice (*Mus musculus*) experimentally infected with myotropic (M) strains of EMCV-1 (Cerutis et al. 1989; Psalla et al. 2006). The myocarditis was minimal or mild, and unlikely to be related to the cause of death, while the pneumonia was more substantial. However, viral load (indicated by Ct values) [Supplementary table S1] was not a predictor of whether or not an animal had histological lesions, as one would expect if the virus were their cause, and the lesions are non-specific, so cannot be definitively attributed to the virus. Pure cultures of *Staphylococcus aureus* were isolated from three organs in one of the affected animals, making a systemic bacterial infection the most likely cause of the histological lesions in this animal. The other two affected animals had negative peritoneal bacterial cultures, but the presence of a pulmonary abscess in one and the history of bacterial endometritis in their dam was suggestive of systemic bacterial infection. There was no evidence of encephalitis in any of the animals examined.

The histological lesions may be attributable to EMCV-2, alternative pathogens or coinfections of EMCV-2 and another agent. It is possible that immunosuppression caused by a systemic bacterial infection has allowed progression of subclinical EMCV-2 infections to clinical disease. A similar effect has been seen with experimental infections of EMCV-1 in mice, where immunocompetent laboratory mice (*Mus musculus*) developed minimal myocarditis, similar to that seen in this study, while T-cell-deficient (nude) mice developed severe lesions (Kishimoto et al. 1985).

This study was not able to conclusively rule out the presence of lesions in all tissues of the hazel dormice from which EMCV-2 RNA was isolated. EMCV-1 strains are reported to cause lesions in a wide variety of murine organs including the heart, brain, lungs and pancreas; the latter is highly susceptible to autolysis and can only reliably be examined in extremely fresh tissue. Autolysis was present in all samples which compromised the ability of histopathology to detect subtle lesions (e.g. neuronal necrosis), but should not have affected the detection of overt lesions such as inflammatory infiltrates (Psalla et al. 2006).

Colocalisation of viral RNA and/or antigen to the observed lesions by in situ hybridisation or immunohistochemistry would provide greater clarity on disease causation (Fredricks and Relman 1996). However, to definitively determine the pathogenicity of EMCV-2, experimental infections are required (Fredricks and Relman 1996). Given a lack of evidence that EMCV-2 has an impact on population numbers, it would not be appropriate to perform such experiments on a vulnerable species like hazel dormice, but model organisms such as laboratory mice would be preferable and allow for better comparison between EMCV-1 and EMCV-2. These experiments would require pure cultures of virus (Gould et al. 2012); the apparently high prevalence in English hazel dormice suggests that they might be a suitable source of tissue for isolation attempts.

EMCV-2 infection was detected in hazel dormice submitted from a wide geographical area across mid- and southern England and that the distribution of EMCV-2-positive cases did not follow a particular pattern; both EMCV-2-positive and EMCV-2-negative cases, for example, were submitted from Devon, Hampshire, Kent, Nottinghamshire and Surrey [Supplementary table S1]. Sample numbers per county were too small to assess relative prevalence, but results suggest that the virus is widely distributed across England. Additionally, sequence phylogeny did not follow a particular pattern; for example, sequence OR001731 was found only in Bedfordshire, but several other sequences such as OR001738 could be found in more than one county (Cornwall, Kent and West Sussex) not geographical linked [Supplementary table S1]. The virus was detected in counties both with and without conservation reintroductions of hazel dormice demonstrating that the virus is not a consequence of translocation of animals and likely circulates as an endemic infection in England. EMCV-2 associated disease is unlikely a primary cause of mortality in the species, based on cases examined histologically in this study. The true impact of disease on free-living populations of small and rare species can, however, be difficult to determine because sick or dead individuals are hard to detect (Wobeser 2007). Therefore, the use of our EMCV-2 PCR protocol, in conjunction with standard diagnostic techniques, is recommended during post-mortem examinations to better understand the impact of EMCV-2 infection on the hazel dormouse population in England.

## Conclusion

This study reports the first detection of EMCV-2 in the hazel dormouse, expanding the host range of this virus and associated whole-genome sequence data. In addition, we provide a RT-qPCR method for detection of EMCV-2 RNA as well as a conventional RT-PCR method for Sanger sequencing of a 379-bp amplicon in the 3D polymerase gene for phylogeny. The high prevalence of EMCV-2 RNA detected in the lungs of English hazel dormice and geographical distribution indicates that EMCV-2 is likely endemic in this species. Histopathological examination of tissues from nine dormice in which EMCV-2-RNA was detected found evidence of lesions, but could not conclusively determine EMCV-2 infection to be the causative agent. Further studies of EMCV-2 pathogenesis would be advised to establish if EMCV-2 infection can cause disease in hazel dormouse and potentially impact the health of the free-living populations in England.

## Supplementary Information

Below is the link to the electronic supplementary material.

Supplementary file1 (DOCX 24 KB)
