# Correction: Prevalence and Molecular Analysis of Encephalomyocarditis Virus-2 in the Hazel Dormouse

**DOI:** 10.1007/s10393-025-01709-x

**Published:** 2025-03-11

**Authors:** Louise Gibson, Tammy Shadbolt, Pranab Paul, Georgina Gerard, Ethan Wrigglesworth, Anthony W. Sainsbury, Helen Donald, Jenny E. Jaffe, Inez Januszczak, Liam D. Fitzpatrick, Caela Burrell, Hannah Davies, Akbar Dastjerdi, Simon Spiro

**Affiliations:** 1 Institute of Zoology, Zoological Society of London, London, NW1 4RY UK; 2 Royal Veterinary College, London, UK; 3 Chattogram Veterinary and Animal Sciences University, Chittagong, Bangladesh; 4 Natural England, London, UK; 5 Tai Chimpanzee Project, Abidjan, Côte d’Ivoire; 6 Natural History Museum, London, UK; 7 UK Health Security Agency, London, UK; 8 Animal and Plant Health Agency-Weybridge, Surrey, UK

**Correction to: EcoHealth 21, 112–122, 2024** 10.1007/s10393-024-01680-z

The article “Prevalence and Molecular Analysis of Encephalomyocarditis Virus-2 in the Hazel Dormouse”, written by Louise Gibson, Tammy Shadbolt, Pranab Paul, Georgina Gerard, Ethan Wrigglesworth, Anthony W. Sainsbury, Helen Donald, Jenny E. Jaffe, Inez Januszczak, Liam D. Fitzpatrick, Caela Burrell, Hannah Davies, Akbar Dastjerdi, and Simon Spiro, was originally published under exclusive license to Springer Science + Business Media, LLC, part of Springer Nature. As a result of the subsequent decision to publish the article under the open access model, the article’s copyright notice was changed on 12 February 2025 to © The Author(s) 2024 and the article is now distributed under a Creative Commons Attribution 4.0 International License, which permits use, sharing, adaptation, distribution and reproduction in any medium or format, as long as you give appropriate credit to the original author(s) and the source, provide a link to the Creative Commons licence, and indicate if changes were made. The images or other third party material in this article are included in the article’s Creative Commons licence, unless indicated otherwise in a credit line to the material. If material is not included in the article’s Creative Commons licence and your intended use is not permitted by statutory regulation or exceeds the permitted use, you will need to obtain permission directly from the copyright holder.

To view a copy of this licence, visit http://creativecommons.org/licenses/by/4.0.

