# Supplementary material for: Prevalence and Molecular Analysis of Encephalomyocarditis Virus-2 in the Hazel Dormouse
Source: Ecohealth. 2024 Apr 23;21(1):112–22. doi: 10.1007/s10393-024-01680-z (PMC11897066; doi:10.1007/s10393-024-01680-z)
Supplement: Supplementary file 1 — Supplementary file1 (DOCX 24 KB) [file 10393_2024_1680_MOESM1_ESM.docx]

**Supplementary Table S1.** Demographic and EMCV-2 RT-qPCR data for 44 hazel dormice testing positive (POS) and negative (NEG) for EMCV-2 infection with cycle threshold (Ct) value. EMCV-2 Genbank accession numbers provided for Sanger sequences of 379 bp amplicons obtained by conventional reverse-transcriptase PCR of the 3D polymerase gene.

| Dormouse | Sex | Age | English county | Reintroduction in county | EMCV-2 qRT-PCR result | Ct value | EMCV-2 Genbank accession number |
| --- | --- | --- | --- | --- | --- | --- | --- |
| 1 | Male | Juvenile | Lincolnshire | Yes | NEG | n/a | n/a |
| 2 | Unknown | Juvenile | Hampshire | No | POS | 19.41 | OR001729 |
| 3 | Male | Juvenile | Cornwall | No | POS | 34.53 | OR001729 |
| 4 | Male | Juvenile | Devon | No | POS | 26.99 | OR001730 |
| 5 | Unknown | Juvenile | Bedfordshire | Yes | POS | 17.66 | OR001731 |
| 6 | Unknown | Juvenile | Bedfordshire | Yes | POS | 25.49 | OR001731 |
| 7 | Unknown | juvenile | Bedfordshire | Yes | POS | 16.83 | OR001731 |
| 8 | Unknown | juvenile | Bedfordshire | Yes | POS | 16.25 | OR001731 |
| 9 | Male | Juvenile | Nottinghamshire | Yes | NEG | n/a | n/a |
| 10 | Unknown | Juvenile | Northamptonshire | Yes | NEG | n/a | n/a |
| 11 | Female | Adult | Devon | No | POS | 22.89 | OR001732 |
| 12 | Male | Juvenile | Nottinghamshire | Yes | POS | 13.23 | OR001733 |
| 13 | Male | Adult | Warwickshire | Yes | POS | 14.38 | OR001734 |
| 14 | Female | Adult | Warwickshire | Yes | POS | 15.58 | OR001735 |
| 15 | Male | Adult | Wiltshire | No | POS | 31.78 | OR001736 |
| 16 | Female | Adult | Devon | No | POS | 18.3 | OR001736 |
| 17 | Female | Adult | Devon | No | NEG | n/a | n/a |
| 18 | Male | Adult | Unknown | Unknown | NEG | n/a | n/a |
| 19 | Unknown | Adult | Surrey | No | NEG | n/a | n/a |
| 20 | Male | Adult | Somerset | No | NEG | n/a | n/a |
| 21 | Female | Adult | Suffolk | Yes | POS | 18.27 | OR001737 |
| 22 | Female | Adult | Cornwall | No | POS | 17.49 | OR001738 |
| 23 | Male | Adult | Gloucestershire | No | POS | 19.25 | OR001739 |
| 24 | Male | Juvenile | Bristol | No | POS | 18.36 | OR001740 |
| 25 | Female | Juvenile | Suffolk | Yes | POS | 15.3 | OR001741 |
| 26 | Female | Juvenile | Devon | No | POS | 36.68 | OR001740 |
| 27 | Female | Adult | Devon | No | POS | 30.47 | OR001741 |
| 28 | Female | Juvenile | Devon | No | POS | 36.85 | OR001740 |
| 29 | Unknown | Juvenile | Kent | No | POS | 31.97 | OR001738 |
| 30 | Unknown | Juvenile | Kent | No | POS | 36.78 | Incomplete consensus sequence |
| 31 | Unknown | Juvenile | West Sussex | No | POS | 37.25 | No consensus sequence |
| 32 | Unknown | Juvenile | West Sussex | No | POS | 19.22 | OR001738 |
| 33 | Unknown | Juvenile | Surrey | No | POS | 16.94 | OR001742 |
| 34 | Unknown | Juvenile | Surrey | No | POS | 18.42 | OR001742 |
| 35 | Unknown | Juvenile | Surrey | No | POS | 37.3 | OR001742 |
| 36 | Unknown | Juvenile | East Sussex | No | POS | 33.08 | No consensus sequence |
| 37 | Unknown | Juvenile | East Sussex | No | POS | 36.26 | OR001742 |
| 38 | Female | Adult | Hampshire | No | NEG | n/a | n/a |
| 39 | Female | Adult | Kent | No | POS | 17.94 | OR001743 |
| 40 | Male | Juvenile | Kent | No | POS | 23.9 | OR001744 |
| 41 | Unknown | Juvenile | Kent | No | NEG | n/a | n/a |
| 42 | Unknown | Juvenile | Kent | No | POS | 37.57 | Incomplete consensus sequence |
| 43 | Unknown | Juvenile | Warwickshire | Yes | POS | 33.73 | OR001742 |
| 44 | Unknown | Juvenile | Warwickshire | Yes | POS | 39.01 | OR001740 |
